# Preparation of Fe@Fe_3_O_4_/ZnFe_2_O_4_ Powders and Their Consolidation via Hybrid Cold-Sintering/Spark Plasma-Sintering

**DOI:** 10.3390/nano14020149

**Published:** 2024-01-10

**Authors:** Amalia Mesaros, Bogdan Viorel Neamțu, Traian Florin Marinca, Florin Popa, Gabriela Cupa, Otilia Ruxandra Vasile, Ionel Chicinaș

**Affiliations:** 1 Physics and Chemistry Department, Technical University of Cluj-Napoca, 103-105 Muncii Avenue, 400641 Cluj-Napoca, Romania; 2 Materials Science and Engineering Department, Technical University of Cluj-Napoca, 103-105 Muncii Avenue, 400641 Cluj-Napoca, Romania; bogdan.neamtu@stm.utcluj.ro (B.V.N.); traian.marinca@stm.utcluj.ro (T.F.M.); florin.popa@stm.utcluj.ro (F.P.);; 3 Department of Science and Engineering of Oxide Materials and Nanomaterials, Faculty of Chemical Engineering and Biotechnologies, National University of Science and Technology Politehnica Bucharest, No 1-7 Polizu Street, S1, 060042 Bucharest, Romania; otilia.vasile@upb.ro

**Keywords:** in situ oxidation, Fe@Fe_3_O_4_ core–shell powder, ZnFe_2_O_4_ nanoparticles, soft magnetic composite, cold-sintering/spark plasma-sintering

## Abstract

Our study is focused on optimizing the synthesis conditions for the in situ oxidation of Fe particles to produce Fe@Fe_3_O_4_ core–shell powder and preparation via co-precipitation of ZnFe_2_O_4_ nanoparticles to produce Fe@Fe_3_O_4_/ZnFe_2_O_4_ soft magnetic composites (SMC) through a hybrid cold-sintering/spark plasma-sintering technique. XRD and FTIR measurements confirmed the formation of a nanocrystalline oxide layer on the surface of Fe powder and the nanosized nature of ZnFe_2_O_4_ nanoparticles. SEM-EDX investigations revealed that the oxidic phase of our composite was distributed on the surface of the Fe particles, forming a quasi-continuous matrix. The DC magnetic characteristics of the composite compact revealed a saturation induction of 0.8 T, coercivity of 590 A/m, and maximum relative permeability of 156. AC magnetic characterization indicated that for frequencies higher than 1 kHz and induction of 0.1 T, interparticle eddy current losses dominated due to ineffective electrical insulation between neighboring particles in the composite compact. Nevertheless, the magnetic characteristics obtained in both DC and AC magnetization regimes were comparable to those reported for cold-sintered Fe-based SMCs.

## 1. Introduction

Soft magnetic composites (SMCs) constitute a significant subclass of the soft magnetic materials field due to their specific capability to produce innovative three-dimensional cores with high magnetic flux density and also due to their potential utilization in various electrical machinery fields [1,2,3,4]. In recent decades, researchers have focused on the enhancement of the magnetic properties of SMCs by studying the effects induced by the thin insulating layer on the surface and by optimizing their elaboration technology [4,5,6,7,8]. An organic insulating layer limits the thermal treatment temperature of SMCs, while an inorganic coating such as Al_2_O_3_, SiO_2_, or ZrO_2_ degrades the saturation magnetization due to the magnetic dilution effect induced by the non-magnetic insulating layer [9,10,11,12,13,14,15]. To overcome these deficiencies, ferromagnetic materials such as ferrite-type systems, Mn-Zn ferrites, Ni-Zn ferrites, and Fe_x_O_y_ (especially Fe_3_O_4_) have been considered as the suitable insulating layer [8,16,17,18,19,20,21,22,23]. Additionally, several studies present the elaboration of SMCs with an inorganic double-insulating layer that enhances heat resistance and enables enhanced magnetic and mechanical performance by high-temperature annealing [4,24,25].

To date, different wet chemical methods such as sol-gel, co-precipitation, hydrothermal or dry strategies, mechanical alloying, and atmosphere oxidation have been developed for the synthesis of composite particles. Even if the dry methods are more facile, their efficiency is lower than the wet chemical approach because it is difficult to form a uniform coating layer on the surface of soft magnetic particles. Additionally, many efforts have been made to control layer thickness and reduce the defects or holes from the interface between the ferrite or oxide layer and metal, drastically diminishing the composite properties [1,2,3,4,5,6]. The melting point, resistivity, and bandgap energy are important characteristics of the coating layer. A high-quality and uniform insulating layer effectively reduces eddy current losses by providing the material with remarkable insulation properties of resistivity and bandgap energy [4]. Recently, Zhang et al. reported that the in situ oxidation method assures the formation of a uniform Fe_3_O_4_ surface coating layer and, moreover, this method facilitates good control over the layer thickness [3]. The co-precipitation method was used by Yi et al. to grow a uniform (NiZn)Fe_2_O_4_ layer on the surface of Fe particles, and the correlation between the structures and magnetic properties was deeply studied [26]. In addition to conventional strategies, the addition of inorganic particles, such as ZrO_2_, Al_2_O_3_, MgO, Fe_3_O_4_, and ZnSO_4_, has been considered to increase the electric resistance and lower the eddy current loss of SMCs [1,15,27,28,29,30]. As chemical methods for the elaboration of inorganic particles, it must be mentioned that sol-gel, precipitation/co-precipitation, or hydrothermal methods are often utilized. Between these chemical strategies, chemical precipitation is simple, less expensive, eco-friendly, allows an easy scale-up method, and assures the formation of homogeneous particles [31,32].

In this study, a step-by-step hybrid strategy was used to fabricate Fe@Fe_3_O_4_/ZnFe_2_O_4_ SMCs. In the first stage, Fe@Fe_3_O_4_ core–shell composites were obtained using an in situ surface oxidation method based on the reaction between Fe powder and O_2_ in a weak acidic media. Additionally, ZnFe_2_O_4_ nanoparticles (ZFnps) were synthetized using a co-precipitation method at room temperature, under pH control, without any thermal treatment. The coating process that we used ensures the complete coating of the Fe particles with a layer of Fe_3_O_4_. However, the thickness of the Fe_3_O_4_ layer is low, leading to the risk of electrical contact between Fe cores. To minimize this risk, a second insulating phase was added. It is worth mentioning that the electrical resistivity of Zn ferrite is approximately 1 Ω∙m and the electrical resistivity of iron ferrite is only 4 × 10^−5^ Ω∙m. In the second stage, the Fe@Fe_3_O_4_ composites and ZFnps were mixed and pressed to obtain a green compact. This compact was subjected to a hybrid cold-sintering/spark plasma-sintering process. No binder was added during the cold-sintering process in order to prevent the negative effects of the organic decomposition. FTIR and XRD investigation techniques have been used to evidence the formation of an oxide layer at the surface of Fe powder and to determine the optimum synthesis parameters for the ZFnps. Fe@Fe_3_O_4_/ZnFe_2_O_4_ soft magnetic composite was prepared via a hybrid cold-sintering/spark plasma-sintering process. The AC and DC magnetic characteristics of the composite are presented and discussed in light of the parameters of the preparation technique.

## 2. Experimental

### 2.1. Materials

Commercial iron powder (NC 100.24 type produced by Höganäs), Zinc acetate dihydrate, Zn(CH_3_COO)_2_·2H_2_O (99.9%, Merck, Darmstadt, Germany), iron (III) nanohydrate, Fe(NO_3_)_3_·9H_2_O (99.9% Merck), sodium hydroxide and NaOH (pellets, Merck), hydrochloric acid, HCl (fuming 37%, Merck), and ethyl alcohol, C_2_H_5_OH (pure, Merck), were used without any further purification.

#### 2.1.1. Synthesis of the Fe@Fe_3_O_4_ Core-Shell Particles

The surface oxidation of Fe particles was achieved by an in situ oxidation method using an oxygen flow. A total of 10 g of iron powder were added under mechanical agitation to a mixture of 0.1 M HCl and 0.1 M KCl solutions heated to 60 °C. An oxygen gaseous phase was introduced into the solution with a flow rate of 20 sccm for 20 min. The obtained particles were separated in a magnetic field, washed several times with distilled water until Cl^−^ ion-free, and dried at 50 °C for 3 h.

#### 2.1.2. Synthesis of ZnFe_2_O_4_ Nanoparticles

The ZnFe_2_O_4_ spinel material was synthetized via the co-precipitation method using the simultaneous addition of reagent techniques (SimAdd) [33]: zinc acetate and iron (III) nitrate as the corresponding starting metal salts and sodium hydroxide as a precipitating reagent. The stoichiometric ratio of Zn^2+^ to Fe^3+^ ions was 1:2 in the initial 0.1 M aqueous solutions. Precipitation was carried out under continuous magnetic stirring, and the mixture of the metal–salt solutions and 1M NaOH aqueous solution were simultaneously added into 1:10 diluted metal precursor solution using a peristaltic pump. Precipitation was carried out under continuous stirring and pH control between 6–10 at room temperature. Each pH value was maintained during the precipitation reaction. The post-precipitation stage consisted of 24 h aging, separation by filtering, washing three times with distilled water, and drying at 80 °C for 8 h.

#### 2.1.3. Elaboration of Fe@Fe_3_O_4_/ZnFe_2_O_4_ Toroidal-Shaped Magnetic Core

First, a mixture of Fe@Fe_3_O_4_ core–shell particles with 5 wt.% ZnFe_2_O_4_ nanoparticles (pH = 7) were prepared using a mortar and pestle. To facilitate the mixing and cold-sintering processes, several milliliters of ethyl alcohol were added to the powder prior to mixing. After 5 min of mixing, the wet powder was placed in a toroidal mold and punched and compacted at a compaction pressure of 500 MPa. The obtained compact (green compact) was subjected to a spark plasma sintering (SPS) process. The sintering experiments were conducted using homemade spark plasma sintering equipment. The process involved maintaining a temperature of 250 °C for 10 min, with a heating rate of 100 °C per minute. A pressure of 25 MPa was applied during sintering, and an argon gas atmosphere was employed for protection. A distinctive feature of our SPS technique was the use of a graphite die partially filled with fine graphite powder, with the sample immersed within it. This graphite powder effectively acted as a pseudo-liquid, ensuring an even distribution of the compaction pressure across the entire surface of the sample, similar to the principles of hot isostatic pressing. The choice of graphite powder was based on its electrical conductivity and anti-friction properties. To apply the compaction pressure of 25 MPa, a set of graphite punches was utilized, maintaining a constant pressure throughout the sintering process. After sintering was complete, the sample was removed from the graZphite die and carefully cleaned to eliminate any adhering graphite particles.

### 2.2. Characterization Techniques

The chemical nature of the Fe@Fe_3_O_4_ core–shell system and ZnFe_2_O_4_ particles was analyzed by Fourier transform infrared spectroscopy (FTIR) using a Tensor 27 Bruker FTIR spectrophotometer (Markham, ON, Canada). The morphology of the cold-sintered compact was investigated via scanning electron microscopy (SEM) and energy dispersive X-ray spectroscopy (EDX). The equipment that was used was a JEOL microscope, type JSM 5600LV, equipped with Ultim Max65 X-ray energy dispersive detectors from Oxford Instruments (Aztech software). Additionally, for the TEM investigation, a Tecnai G2 F30 S-TWIN transmission electron microscope at an accelerating voltage of 300 kV was used. The structural characterization of the core–shell particles (Fe/Fe_3_O_4_) and ZnFe_2_O_4_ nanoparticles were made by X-ray diffraction (XRD). The equipment used was an INEL Equinox 3000 diffractometer. The wavelength used was the one characteristic of Co, Kα radiation (λ = 1.7903 Å). The mean crystallite size was calculated by the Scherrer method.

The magnetic characteristics of the toroidal-shaped magnetic core were assessed using a computer-controlled Remagraph–Remacomp C-705 hysteresisgraph manufactured by Magnet-Physik Dr. Steingroever GmbH (Hannover, Germany), Hannover, Germany. The magnetic characteristics were measured in both static (DC) and dynamic (AC) magnetization regimes. For DC measurements, the maximum field applied was 8.5 kA/m. AC magnetic measurements were carried out under a constant induction value (B_max_) of 0.1 T, covering a frequency range from 50 Hz to 10 kHz. The electrical resistivity of the toroidal-shaped magnetic core was measured using a four-point probe measurement setup.

## 3. Results and Discussion

### 3.1. Fe@Fe_2_O_4_ Core-Shell Powders

The chemical processes behind the formation of the oxidized layer at the surface of iron particles were studied and explained based on the iron corrosion theory [3]. Oxidation of the iron surface at high temperatures and under acidic media is favored by the presence of oxygen. The first process involved the transfer of electrons due to the oxidation of the Fe atom with the formation of Fe^2+^, followed by a reduction in H^+^ with the formation of H_2_. Concurrently, the water ionization process took place with the formation of OH^−^ and H^+^ ions. Additionally, the oxidation of Fe^2+^ to Fe^3+^ was possible due to the presence of water and oxygen and all ionic entities chemically reacted with the formation of Fe_3_O_4_. The oxidation of Fe particle surface can be explained based on the following chemical reactions [3]:

Fe − 2e^−^ → Fe^2+^,

H_2_O → H^+^ + OH^−^,

H^+^ + 2e^−^ → H_2_,

Fe^2+^ + 2OH^−^ → Fe(OH)_2_,

2Fe(OH)_2_ + ½ O_2_ + H_2_O → 2Fe(OH)_3_,

Fe(OH)_2_ + 2Fe(OH)_3_ → Fe_3_O_4_ +4H_2_O.

**Figure 1:**
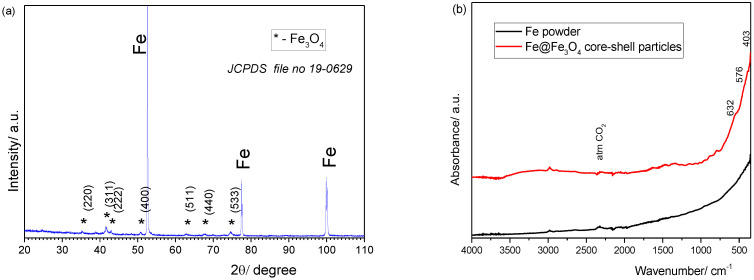
XRD pattern (**a**) and FTIR spectra (**b**) of Fe powder and the Fe@Fe_3_O_4_ system after 20 min in situ oxidation of iron powder.

In our work, the formation of the oxide layer on the surface of the Fe particles was investigated by FTIR and XRD measurements, as shown in Figure 1. The XRD pattern of the Fe@Fe_3_O_4_ system is presented in Figure 1a. There are three main diffraction lines at 52.5°, 77.4°, and 99.8°, corresponding to the (110), (200), and (211) planes of the α-Fe powder (JCPDS file no. 06-0696). Low-intensity additional lines (*) can be noticed, corresponding to the (220), (311), (400), (422), (511), (440), and (533) planes of the Fe_3_O_4_ cubic spinel structure (JCPDS file no. 19-0629). Additional peaks representing supplementary phases were not observed. Additionally, our oxidized powder presented a significant color change from silver to black, indicating the emergence of the Fe_3_O_4_ coating on the surface. The mean crystallite size of the Fe_3_O_4_ layer, calculated using the (311), (440), and (533) reflections, was 20 ± 2 nm.

The FTIR spectra of the iron powder before and after in situ oxidation are shown in Figure 1b. The three intense peaks observed at 403 cm^−1^, 576 cm^−1^, and 632 cm^−1^ are attributed to the vibration modes associated with the Fe-O bonds in the crystalline lattice of Fe_3_O_4_. Low-intensity bands can be observed at approximately 1630 cm^−1^ and 340 cm^−1^ related to OH-bending and OH-stretching in the hydroxyl group [34]. From the above-discussed results, it can be concluded that a Fe@Fe_3_O_4_ composite system was formed.

**Figure 2:**
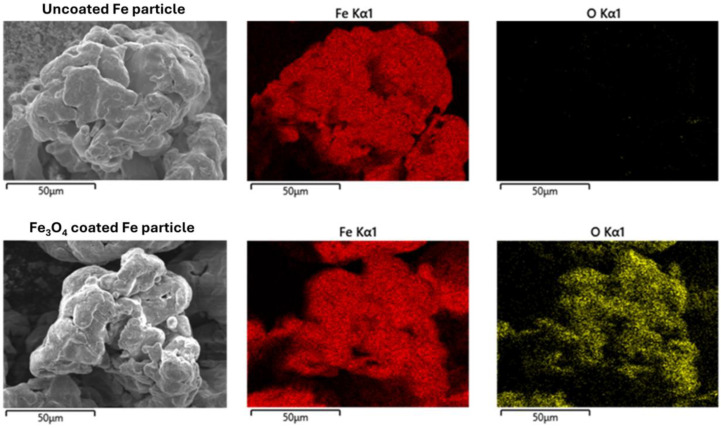
SEM-EDX analysis of the uncoated and Fe_3_O_4_-coated particles.

SEM-EDX investigations were performed in order to present the morphology of the coated and uncoated Fe particles, as shown in Figure 2. It can be noticed that a uniform layer of oxide was formed by the chemical coating of Fe particles. As revealed by the XRD investigation, the oxide layer consists of a Fe_3_O_4_ phase.

### 3.2. ZnFe_2_O_4_ Nanoparticles

It is generally accepted that the chemical mechanism is crucial in studying and controlling the precipitation/co-precipitation process by SimAdd techniques [34,35]. The formation of ZnFe_2_O_4_ product during the co-precipitation processes of Zn^2+^ and Fe^3+^ ions with NaOH is governed by the following chemical reaction:

1Zn^2+^ + 2CH_3_COO^−^ + 2Fe^3+^ + 3NO_3_^−^ + 8NaOH → Zn(OH)_2_ + 2Fe(OH)_3_ + 8Na^+^ + 2CH_3_COO^−^ + 3NO_3_^−^,

Zn(OH)_2_ + 2Fe(OH)_3_ → ZnFe_2_O_4_ + 4H_2_O.

**Figure 3:**
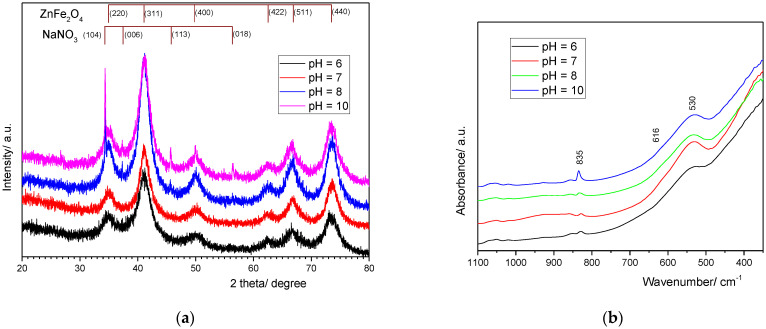
XRD patterns (**a**) and FRIR spectra (**b**) of the ZnFe_2_O_4_ samples synthetized at different pH values of precipitation media.

The structural characteristics of ZnFe_2_O_4_ samples obtained at different pH values of the precipitation media were determined by XRD measurements, as shown in Figure 3a. The diffraction patterns reveal the presence of the reflection lines corresponding to the spinel cubic structure of ZnFe_2_O_4_ for the four samples (JCPDS card no 22-1012). A supplementary phase attributed to the NaNO_3_ crystalline structure (JCPDS card no. 36-1474) can be noticed for the samples obtained when the precipitation media are under basic pH conditions. This can be explained by the addition of a large amount of the precipitating agent, which leads to the formation of sodium salts, e.g., acetates and nitrates, as the secondary products, as reaction (2) suggests. The washing procedure assumes the removal of these supplementary products from the solution that contains a large amount of sodium salts. Since sodium acetate presents higher solubility in water, it is removed first, and then sodium nitrate can partially remain crystallized on the ZnFe_2_O_4_ surface. The presence of nitrate ions in the samples obtained in basic media was confirmed by FTIR spectra, Figure 3b, through the presence of a low-intensity peak from 835 cm^−1^, characteristic for the symmetric stretching vibration of the N-O bond in NO_3_^−^ [36].

Additionally, the XRD measurements are indicative of the distribution of the Zn^2+^ to Fe^3+^ ions in the cubic spinel structure between tetrahedral and octahedral positions. It is well known that a normal cubic spinel structure is obtained when Zn^2+^ cations occupy tetrahedral positions. On the contrary, an inversed spinel structure is formed when Zn^2+^ cations prefer octahedral sites, and in terms of the lattice parameter, a value lower than 8.44 Å, characteristic for Fe_3_O_4_, is obtained [37]. The calculated lattice parameters for the four samples (Table 1) suggest that an inversed spinel structure is formed when the precipitation takes place at neutral pH. A weak acidic media (pH = 6) or a basic pH domain (pH = 12) leads to an increase in lattice parameters, suggesting that a normal spinel structure is formed. Additionally, broad XRD patterns reveal that the obtained ferrite particles are nanosized. The mean crystallite size, calculated through the Scherrer method applied to the (311), (511), and (440) reflections (Table 1), suggests that a neutral pH promotes the acceleration of the growth phase.

**Figure 4:**
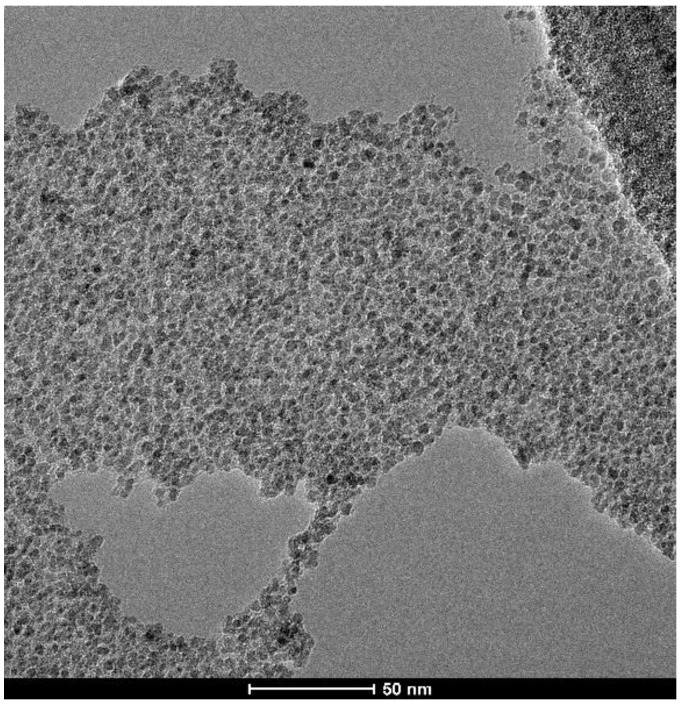
TEM image for ZnFe_2_O_4_ particles obtained at neutral pH.

The morphological characteristics for ZnFe_2_O_4_ particles obtained at neutral pH (pH = 7) were investigated by TEM and are presented in Figure 4. The TEM image illustrates the formation of spherical-shaped, small, and agglomerated zinc ferrite nanoparticles. The ZnFe_2_O_4_ particle size, as revealed by TEM, is approximately 5–6 nm. This indicates that the ZnFe_2_O_4_ nanoparticles consist of one crystallite if the TEM data are correlated with the XRD data.

### 3.3. Hybrid Cold-Sintered/Spark Plasma-Sintered Soft Magnetic Composite

**Figure 5:**
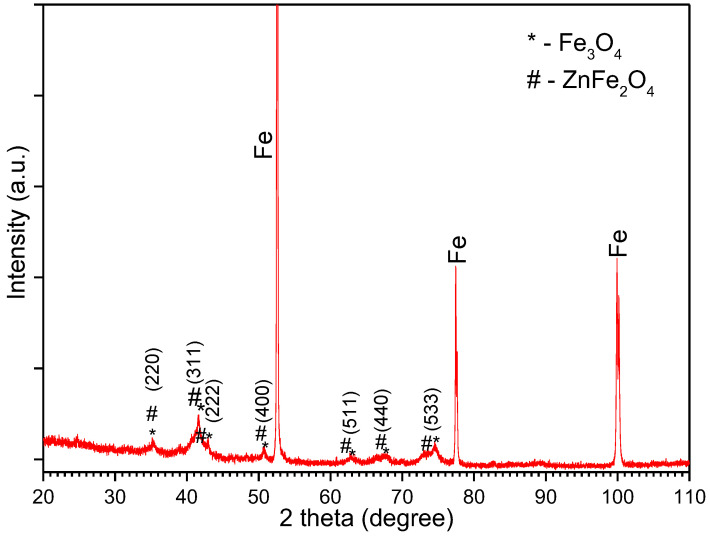
X-ray diffraction pattern of the compact obtained by hybrid sintering CS-SPS.

Figure 5 show the X-ray diffraction pattern obtained on the hybrid sintered CS-SPS for the composite compact Fe/Fe_3_O_4_/ZnFe_2_O_4_. It can be noticed that the sintering process led to the maintaining of the phases present in the material before sintering. According to the XRD analysis, no reaction occurred between phases during CS-SPS sintering. Fe_3_O_4_ and ZnFe_2_O_4_ have the same crystallographic structure and, therefore, their diffraction maxima almost entirely overlap at lower diffraction angles.

**Figure 6:**
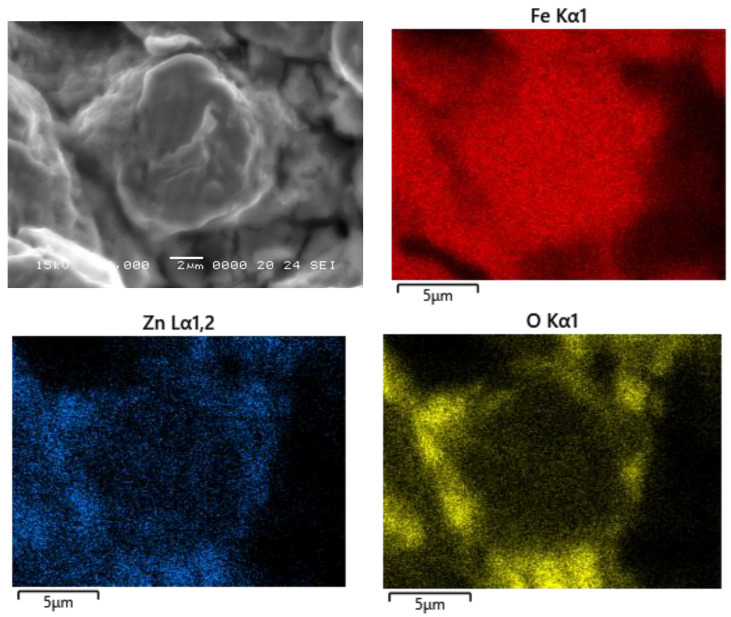
SEM image and elemental distribution maps (EDX) of Fe, Zn, and O on the cross-section of the composite compact prepared via a hybrid cold-sintering/spark plasma-sintering process.

The SEM-EDX investigation of cold-sintered compact is presented in Figure 6. Analyzing the elemental distribution maps of Zn and O in the cross-section of the sample, it can be concluded that the ZnFe_2_O_4_ nanoparticles are distributed on the surface of the Fe@Fe_3_O_4_ particle, forming a quasi-continuous insulating layer. The uniformity of the Fe_3_O_4_ coating on the surface of Fe particles cannot be evaluated by analyzing the cross-section of the cold-sintered sample since the coating contains only Fe and O atoms, and these elements are also contained by the Fe particles and ZnFe_2_O_4_ nanoparticles.

**Figure 7:**
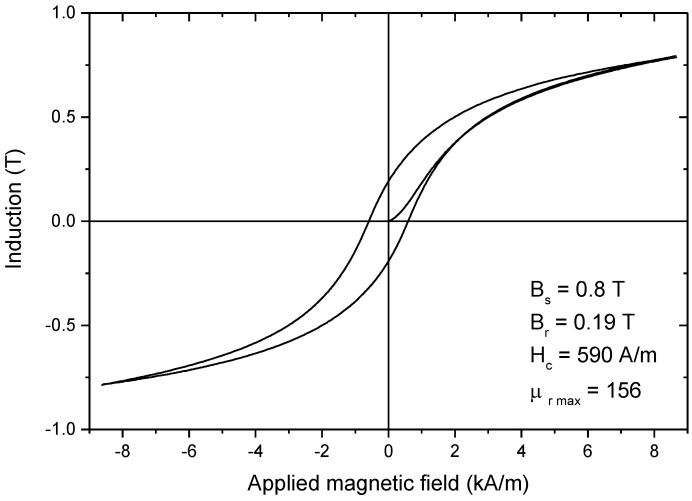
DC hysteresis loop of the composite compact prepared via a hybrid cold-sintering/spark plasma-sintering process.

The DC hysteresis loop of the composite compact is presented in Figure 7. The soft magnetic behavior of the composite magnetic core, prepared via a hybrid cold-sintering/spark plasma-sintering process, can be observed by analyzing the shape of its hysteresis curve and the values of saturation induction (B_s_), remanence (B_r_), coercivity (H_c_), and maximum relative permeability (µ _rmax_) provided in Figure 4. It can be noted that applying a magnetic field of 8.5 kA/m results in an induction of 0.8 T. The obtained induction value is comparable to other values reported in the literature. For example, in the case of cold-sintered compacts based on Fe powder coated with ZnO, depending on the amount of ZnO used (10–20 wt.%), a saturation induction of 0.66–0.83 T was reported [38]. The coercivity of the composite compact presented in this study (590 A/m) is higher than the coercivity reported for the above-mentioned compacts based on Fe powder coated with ZnO (469–490 A/m). The increased coercivity can be explained by the influence of the coating technique on the magnetic characteristics of the powders. The ZnO coating was prepared by immersing the Fe powder in a solution containing ZnO nanoparticles and acetic acid. No significant reaction between the base material and the coating was reported. On the other hand, the Fe_3_O_4_ coating was prepared via an in situ oxidation method, which is a conversion-coating technique (the superficial metallic layer of the particles is converted into an oxide layer). An interface between the core and the shell of the particle is created during the in situ oxidation process. This interface contains a large number of pinning centers that hinder the displacement of the magnetic domain walls, thus increasing the coercivity of the powders and, consequently, the coercivity of the compact. The presence of these pinning centers also leads to a decrease in the value of the maximum relative permeability of the compact (156) compared to the value obtained in the case of cold-sintered compacts based on Fe powders coated with ZnO (174) [38].

**Figure 8:**
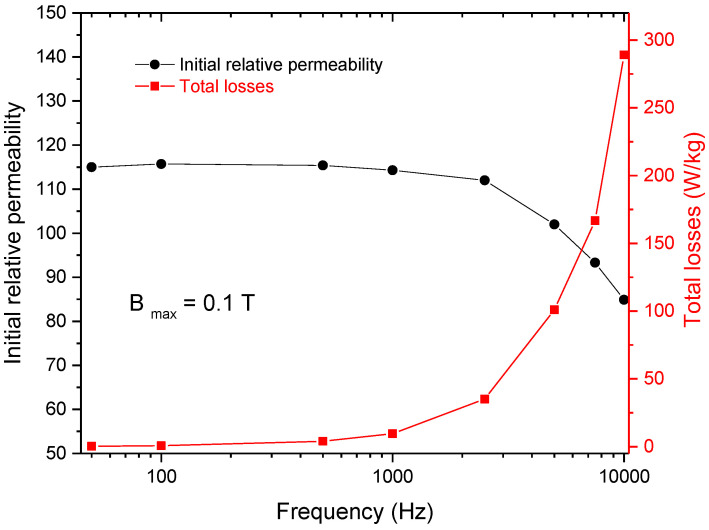
Initial relative permeability and total core losses vs. frequency for the hybrid cold-sintered/spark plasma-sintered compact.

The variation in the initial relative permeability and total core losses vs. frequency for the hybrid cold-sintered/spark plasma-sintered compact is presented in Figure 8. The value of the initial relative permeability of the compact is practically constant up to the frequency of 2 kHz. At higher frequencies, a significant decrease in the value of the permeability of the compacts is noticed. Generally, when a decrease in the value of the initial relative permeability of the SMCs is noticed, it indicates the excessive development of eddy currents in the sample. The eddy currents that circulate in the cross-section of the sample create a magnetic field that will oppose the applied magnetizing field thus reducing the permeability of the sample. The excessive development of the eddy currents in the sample is also proven by the more abrupt increase in the total core losses of the sample for frequencies higher than 1 kHz. Similar results were also reported in the case of cold-sintered SMCs based on Fe powder coated with 10 or 20 wt.% ZnO [38].

To have a better understanding of the main dissipative mechanism of our sample, a loss separation procedure was applied. The total losses of the sample (*P_t_*) are composed of hysteresis losses (*P_h_*), eddy current losses and excess losses (*P_e_*) [39].

(1) $$P_{t}=P_{h}+P_{e}+P_{exc}.$$

The contribution of the excess losses was neglected in this study since their contribution to the total losses is significant only at very high frequencies and very low induction. The eddy current losses are composed of intraparticle eddy current losses and interparticle eddy current losses. The total losses provided by Equation (4) can be written as:

(2) $$P_{s}=P_{h}+P_{e}^{inter}+P_{e}^{intra}.$$

Hysteresis losses are calculated by the extrapolation to 0 Hz of total losses. According to the literature, the formulas for the intraparticle and interparticle eddy current losses are [40]:

(3) $$P_{e}^{intra}=\frac{({\pi dB_{m})}^{2}}{20R_{Fe}\rho_{Fe}}f^{2},$$

and

(4) $$P_{e}^{inter}=\frac{({\pi d_{eff}B_{m})}^{2}}{\beta R_{s}\rho_{s}}f^{2},$$

where *d* is the diameter of the particles, *B_m_* is the maximum induction, *f* is the frequency, *R_Fe_* is the specific resistivity of Fe, *ρ_Fe_* is the specific density of the Fe, *d_eff_* is the effective dimension for eddy current, *R_s_* is the electrical resistivity of the sintered sample, and *ρ_s_* represents the density of the sintered toroidal compacts. The *β* coefficient can be calculated using the following relation:

(5) $$\beta =\frac{6}{1-0.633\frac{w}{h}\tanh\left(1.58\frac{h}{w}\right)},$$

where *w* is the width of the rectangle, and *h* is the rectangle height (the cross-section of the sample is a rectangle).

**Figure 9:**
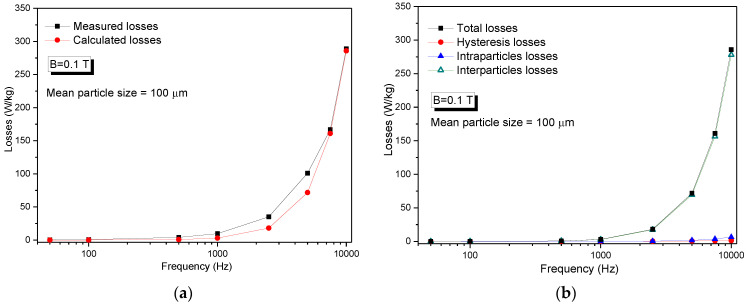
Results of loss separation procedure applied to the hybrid cold-sintered/spark plasma-sintered compact. (**a**) Comparison between measured and calculated losses. (**b**) Decomposition of losses in hysteresis, intraparticle, and interparticle losses.

The particle size that is used for loss separation is the median size provided by the producer of the powder; in our case, the mean particle size is 100 μm. Figure 9a presents the comparison between the measured losses and the losses calculated with this model. Good agreement between the measured and calculated losses, according to Equation (5), is obtained.

Analyzing Figure 9b, it can be observed that the value of hysteresis and dynamic losses (intraparticle and interparticle) are comparable up to the frequency of 1 kHz. At frequencies higher than 1 kHz, an excessive increase in the interparticle eddy current losses is observed. The interparticle eddy current losses become the main part of the total losses of the composite. For example, at a frequency of 10 kHz, the hysteresis losses represent approximately 0.5% of total losses, and the intraparticle eddy current losses (the eddy currents that are restrained inside individual particles) represent 2.3% of total losses of the compact. We recall that the interparticle eddy currents are currents that develop across the boundary of the particles. The fact that, for frequencies higher than 1 kHz, this type of loss is dominant indicates that the particles are not properly insulated from each other and/or the layer thickness is not large enough to offer good electrical resistivity. Another important aspect that is necessary to be considered is that the electrical resistivity of the magnetite (iron ferrite) is much lower compared to the one of zinc ferrite (4 × 10^−5^ Ω∙m for iron ferrite and 1 Ω∙m for zinc ferrite [40]). Although the magnetite covers the iron particles well, it cannot confer a very high electrical resistivity. The large increase in interparticle losses can be associated with the inhomogeneous insulation offered by zinc ferrite nanoparticles, which possess larger electrical resistivity as compared to magnetite. Special attention must be paid to increasing the quality of the insulating coating in such a way that during the sintering procedure, the insulating coating will not be destroyed.

## 4. Conclusions

In summary, this work allowed us to optimize the synthesis conditions for in situ oxidation of Fe particles and co-precipitation of ZnFe_2_O_4_ nanoparticles. Fe@Fe_3_O_4_/ZnFe_2_O_4_ SMC was obtained via hybrid cold-sintering/spark plasma-sintering. ZnFe_2_O_4_ nps were obtained using co-precipitation, a scalable method, and directly from the precipitation solution, without any thermal treatment.

XRD and FTIR measurements evidenced both the formation of an oxide layer at the surface of Fe powder and the nanosized nature of ZFnps. The SEM-EDX investigations highlighted that the oxidic phase of our composite (Fe_3_O_4_ and ZnFe_2_O_4_) was distributed on the surface of the Fe particles, forming a quasi-continuous matrix.

The DC magnetic characteristics of the composite compact were B_s_ = 0.8 T, H_c_ = 590 A/m, and μ _rmax_ = 156. The AC magnetic characterization highlighted that for frequencies higher than 1 kHz, interparticle eddy current losses were the main part of the losses as a result of insufficient insulation of neighbouring particles in the composite compact. However, the magnetic characteristics determined in both DC and AC magnetization regimes were comparable to other magnetic characteristics reported for cold-sintered Fe-based SMCs.

## Figures and Tables

**Table 1:** Lattice parameter (a) and crystallite mean size (D) calculated from the XRD patterns for the samples synthesized at different pH values for the precipitation process.

ZnFe_2_O_4_ Sample	a (nm)	D (nm)
pH6	8.4522	3.8
pH7	8.4389	5.2
pH8	8.4498	5.1
pH10	8.4636	4.7

## Data Availability

The data presented in this study are available on request from the corresponding author.
